# Deep joint learning of pathological region localization and Alzheimer’s disease diagnosis

**DOI:** 10.1038/s41598-023-38240-4

**Published:** 2023-07-19

**Authors:** Changhyun Park, Wonsik Jung, Heung-Il Suk

**Affiliations:** 1 Department of Brain and Cognitive Engineering, Korea University, Seoul, 02841 Republic of Korea; 2 Department of Artificial Intelligence, Korea University, Seoul, 02841 Republic of Korea

**Keywords:** Machine learning, Neurodegenerative diseases

## Abstract

The identification of Alzheimer’s disease (AD) using structural magnetic resonance imaging (sMRI) has been studied based on the subtle morphological changes in the brain. One of the typical approaches is a deep learning-based patch-level feature representation. For this approach, however, the predetermined patches before learning the diagnostic model can limit classification performance. To mitigate this problem, we propose the BrainBagNet with a position-based gate (PG), which applies position information of brain images represented through the 3D coordinates. Our proposed method represents the patch-level class evidence based on both MR scan and position information for image-level prediction. To validate the effectiveness of our proposed framework, we conducted comprehensive experiments comparing it with state-of-the-art methods, utilizing two publicly available datasets: the Alzheimer’s Disease Neuroimaging Initiative (ADNI) and the Australian Imaging, Biomarkers and Lifestyle (AIBL) dataset. Furthermore, our experimental results demonstrate that our proposed method outperforms the existing competing methods in terms of classification performance for both AD diagnosis and mild cognitive impairment conversion prediction tasks. In addition, we performed various analyses of the results from diverse perspectives to obtain further insights into the underlying mechanisms and strengths of our proposed framework. Based on the results of our experiments, we demonstrate that our proposed framework has the potential to advance deep-learning-based patch-level feature representation studies for AD diagnosis and MCI conversion prediction. In addition, our method provides valuable insights, such as interpretability, and the ability to capture subtle changes, into the underlying pathological processes of AD and MCI, benefiting both researchers and clinicians.

## Introduction

Alzheimer’s disease (AD) is a type of neurodegenerative brain disorder characterized by an irreversible and progressive loss of neurons^1^. This degenerative disorder is also considered the most common cause of dementia^2^, which is commonly accompanied by such symptoms as memory loss and progression toward long-term impairment of cognitive functioning. These symptoms are not manifest in the early stages of AD, and the symptoms gradually worsen without being recognized as the condition^3^. Concretely, AD primarily progresses through a prodromal stage referred to as mild cognitive impairment (MCI). Thus, in the past few decades, numerous studies^4–6^ have focused on both accurately classifying the AD group from the normal control (NC) group and predicting the MCI conversion/transition to detect the early stages of AD. Typically, the MCI conversion prediction task is to distinguish between stable MCI (sMCI) and progressive MCI (pMCI) based on the risk of AD progression. These studies on AD diagnosis and its early detection can help identify high-risk cohorts for better treatment planning and to further improve the quality of life^7^. Although the symptoms that appear in AD are not manifested in the early stages, the biological processes underlying the disease are present for decades before the symptoms occur^8,9^. Therefore, neuroimaging data, such as magnetic resonance imaging (MRI), have been employed for examining neurodegeneration, diagnosing AD, and detecting its early stages^10,11^. Specifically, automated computer-aided diagnosis (CAD) based on structural MRI (sMRI) has been attracting attention as promising studies^12–14^ to capture and utilize the characteristics of brain atrophy, which is one of the proximate substrates of cognitive impairment in AD. In addition, the presentation of local clinical evidence for the diagnostic result has recently been emphasized for clinical application^15,16^.

Most of the conventional CAD methods were multi-step pipelines consisting of the region of interest (ROI) selection, feature extraction, and classification. However, convolutional neural networks (CNNs) exhibited superior performance in image recognition by effectively learning the local feature extraction and image classification in an end-to-end manner. This effectiveness and efficiency have let CNNs applied in various domains, including the medical field^17–19^. Likewise, three-dimensional (3D) CNN-based AD diagnosis models can filter out task-oriented structural patterns such as brain atrophy from 3D sMRI scans. Therefore, recent studies on AD diagnosis^13,20–22^ have concentrated on CAD systems in relation to sMRI scans with 3D CNNs. Most existing 3D CNN-based AD diagnostic models^7,13,17,22–26^ can be categorized into two classes according to the input type. One option is to take a 3D whole-brain image as input. The other option is to take a bag of instances in the multiple instance learning (MIL) framework, where each entire 3D brain image is regarded as a bag, and the 3D patches extracted from the bag are treated as instances. In the case of taking a bag of instances as input, various approaches^27–29^ for patch-level feature representation have attempted to more efficiently characterize local structural changes induced by AD. In addition, a novel feature representation using the CNN model^22,25,26,30,31^ has recently been proposed. These approaches are intended to extract local and subtle structural changes by allowing models to extract features with an upper bound on the patch size. Conventional methods^11,32^ generally assign the class label of an image to all patches extracted from the corresponding image to extract patch-level feature representation. However, the class labels for each patch are ambiguous because not all patches extracted from patients’ brains include changes associated with pathology^33^. Since the proportion of AD-induced brain atrophy might vary according to the individual patient, in this circumstance, weakly supervised learning strategies, such as MIL, have been adopted^25,29^. In MIL, there are two topics of attracting researchers. The first is to provide the rationale for decision-making, and the second is to detect key instance that trigger the bag label. As for the rationale for decision-making, the existing methods^34,35^ have poor instance-level accuracy and are inconsistent in MIL methods at the instance level^36^. One of the promising reasons for this is that MIL models may detect only the most abnormal parts of the image where multiple abnormalities are present^37^. To alleviate the issue,^38^ introduced an attention-based MIL. The attention mechanism was used to determine instances that trigger the bag label, called key instances^39^. Compared to the attention-based MIL, discriminative brain-region localization in AD studies can be considered a type of key instance detection to localize brain regions where the bag label is triggered.

In AD diagnostic research, the MIL method considers a given brain MRI scan as a bag and treats the 3D patches that make up the bag as instances. The simplest method to define the 3D patches in AD diagnosis is to use all of the patches over the brain. However, the number of patches that can be extracted from 3D high-resolution brain images is too large. In addition, including numerous patches not associated with AD can lead to a low proportion of instances containing AD pathological changes in bags labeled as the AD class. This method can cause serious class imbalance problems and degrade performance for many real-world problems^40,41^. Therefore, localizing the brain regions and extracting patches from these regions are important and challenging problems. Although existing studies have addressed these challenges based on data-driven approaches^25,27,29,42^, the predetermined area to extract patches can lead to various limitations. One of them is that the dependency between AD-associated brain-region localization for patch extraction and the diagnostic model training is overlooked. First, it may lead to a low proportion of instances containing AD pathological changes in bags labeled as the AD class. Second, it can limit the opportunity to explore potential biomarkers in the learning diagnosis model. These can result in suboptimal classification performance. In addition, hyperparameters for patch extraction eventually cause time-consuming hyperparameter exploration or optimization procedures by iterating the diagnostic model training according to numerous hyperparameter combinations. To alleviate issues arising from the independence of patch extraction and diagnostic model training, a hierarchical fully convolutional network (H-FCN) was proposed for joint atrophy localization and disease identification^26^. Although a novel discriminative brain-region localization approach has been introduced for patch extraction, the fundamental problem caused by brain-region predetermination remains.

**Figure 1:**
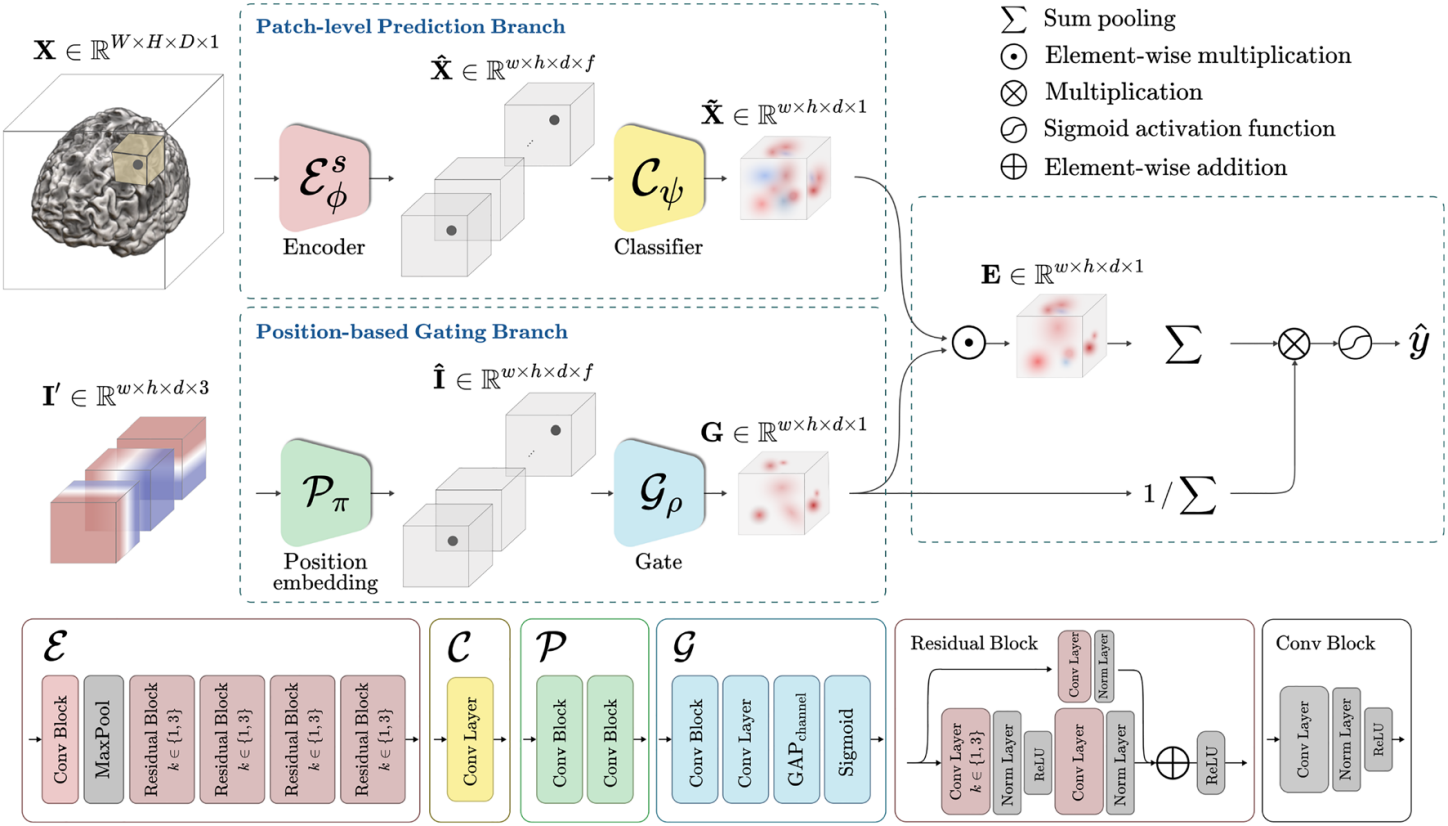
Illustration of the proposed PG-BrainBagNet, including the encoder, classifier, position embedding, and gate network. Structural and position information is processed in two separate branches, given a magnetic resonance imaging scan and position indicator. The outcomes from the two branches are combined to represent patch-level class evidence and image-level disease probability.

In the end, we propose the BrainBagNet with a position-based gate (PG-BrainBagNet) for joint learning of pathological region localization and disease diagnosis. As illustrated in Fig. 1, the proposed method starts with two branches, called the patch-level prediction branch and the position-based gating branch. Given an MRI scan, the patch-level prediction branch extracts local features from a specific receptive field size (i.e., 3D patches) and produces patch-level responses. Then, given the position information, the position-based gating branch identifies the brain regions where the class-discriminative responses consistently appear across subjects. By leveraging the soft region proposals generated by the position-based gating branch, patch-level responses obtained in the patch-level prediction branch are converted to patch-level class evidence, which is transparently aggregated to determine an image-level response. Transparent aggregation for the image-level response alleviates the difficulty of interpretation due to global and nonlinear interactions. In our proposed method, the patch-level class evidence is trained and inferred without the need for local annotation. Instead, we utilize image-level weak annotation to guide the learning process. Taking into account this underlying concept, our approach can be considered a form of multiple-instance learning. We strictly evaluated the effectiveness of the proposed method in both the Alzheimer’s Disease Neuroimaging Initiative (ADNI) dataset and the Australian Imaging Biomarkers and Lifestyle Study of Ageing (AIBL) dataset. In addition, we conducted extensive ablation studies in both AD versus NC and sMCI versus pMCI binary classification tasks, called AD diagnosis and the MCI conversion prediction task, respectively. The proposed method outperformed the comparison methods in both AD diagnosis and MCI conversion prediction tasks. We identified the discriminative brain regions and patch-level class evidence in a weakly supervised learning manner and analyzed the changes according to patch size.

**Figure 2:**
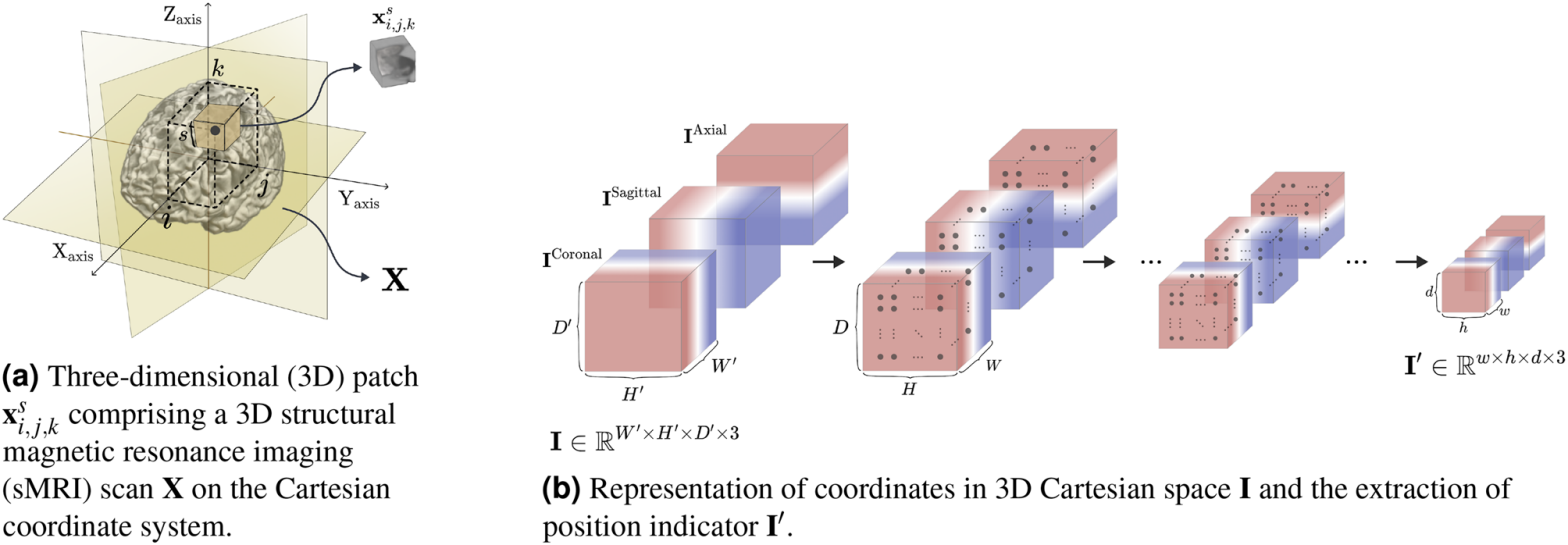
(**a**) Three-dimensional (3D) patch $\textbf{x}^{s}_{i,j,k}$ comprising a 3D structural magnetic resonance imaging (sMRI) scan $\textbf{X}$ on the Cartesian coordinate system. (**b**) Representation of coordinates in 3D Cartesian space $\textbf{I}$ and the extraction of position indicator $\textbf{I}'$. Illustration of (**a**) three-dimensional (3D) patch extracted in a specific position and size and (**b**) the position indicator extracted from the coordinates in 3D Cartesian space.

## Related works

### CNN-based Alzheimer’s disease diagnosis

The development of deep learning methods, including CNN, has efficiently addressed multistep pipelines for handcrafted feature generation/extraction and logistic regression by training a model in an end-to-end manner^17^. Thus, studies on the accurate AD diagnosis based on the 3D CNN are underway by taking 3D whole-brain images as input^13,20^. In particular, various architectures have been proposed for accurate AD diagnosis^9,13,17,23,24^. Among all,^13^ demonstrated the changes in disease identification performance according to various factors, such as the normalization layer, kernel size, network architecture width, and patient age. For network architecture, the model introduced in^13^ was designed to be capable of learning subtle differences in the brain by avoiding early spatial downsampling and limiting the size reduction of feature maps during low-level feature extraction steps. Afterward, the attention mechanism gradually became popular and widely employed in the CNN-based image recognition model to better explain network behavior and generate more discriminative feature representations^19,43,44^.

In terms of AD diagnosis and analysis,^21,23^ introduced an attention-based 3D convolutional network for disease identification and biomarker exploration. The network architecture was designed based on the ResNet^45^, and the attention module was embedded in the middle of the network. By taking the extracted local features as input, the attention module produced spatial weights. As described in^21^, the goal of the attention module is to represent the regional importance during end-to-end training. In backward propagation, the produced attention could work as a gradient filter. Recently,^31^ devise a method that combined multiview-slice attention with 3D CNN to effectively analyze MRI data. By focusing on multiple views of the slice brain image and leveraging the 3D information, the model enables to improve the AD diagnosis performance. As the early AD stages could only be identified using subtle local pathological cues, patch-level feature representations have been investigated to capture subtle local pathological changes more efficiently. Given that the early stages of AD rely on subtle local pathological cues for identification, some researchers have explored patch-level feature representations to enhance the detection of these subtle changes. However, since only a limited number of brain regions may contain relevant cues for disease identification, an additional discriminative patch extraction procedure was necessary before analysis. In previous studies, a common initial step for extracting discriminative patches involved leveraging prior anatomical knowledge and utilizing statistical approaches to derive the discriminative probability^33^. Patch extraction has been improved through resampling schemes using Elastic Net^29,46^. Recently, a landmark discovery algorithm for AD diagnosis was introduced for discriminative patch extraction^25^. The algorithm started with a multivariate statistical test on training images generated using nonlinear registration. A *p*-value map was obtained from the template space. Based on the *p*-value map, landmarks were determined based on the size and number of patches and the minimum distance between patches. Patches extracted from the landmark were used for diagnosis model training. 3D CNN-based feature extractors were configured as the number of determined landmarks to extract features from each patch located in the landmark. The following fully connected layers employed concatenated patch-level representations for bag-level prediction.

However, previous studies conducted discriminative region localization and patch extraction independent of the diagnostic model, which may lead to suboptimal diagnostic performance^26^. To alleviate this limitation,^26^ proposed a hybrid loss and pruning strategy based on the H-FCN. The proposed model extracted multiscale feature representations (i.e., patch-, region-, and subject-level features) by employing CNN. This hierarchical construction of the network architecture allows the trained model to identify the most informative patches and regions through hybrid loss. The hybrid loss was designed by gathering patch-, region-, and image-level loss for diagnostic model training and ranking the discriminative capacity of the corresponding location. Pruning less discriminative areas identified by hybrid loss could improve diagnostic results. However, the hybrid loss was defined by considering all patch- and region-level features belonging to the patient’s MRI images as positive samples, although not all patches and regions would necessarily be affected by AD. In addition, this approach still relies on predetermined landmarks for better classification performance in the initial stage, and the pruning approach cannot consider potential patch-level biomarkers not previously extracted as candidates.

More recently, a hybrid network (HybNet)^22^ was proposed to improve the H-FCN, which considers both global and local structural information. Specifically, two branches were constructed: the global branch (GB) and the local branch (LB). The subject-specific and intersubject-consistent discriminative region localization approaches were applied in the GB and LB, respectively. Both localizations were obtained using a pre-trained fully convolutional network (FCN) backbone. The FCN backbone was trained for weakly supervised object localization (WSOL) and capable of generating class activation maps^47^. They utilized disease attention maps (DAMs) derived from the class activation map outputs to represent subject-specific brain regions associated with AD. Furthermore, the mean of the DAMs is calculated by averaging the DAMs generated from a substantial number of samples in the training dataset. Leveraging the pre-computed localization results, the GB and LB models were trained. The GB utilized DAMs as spatial attention, representing subject-specific discriminative brain regions. On the other hand, LB was trained using patches extracted from intersubject-consistent discriminative brain regions, as indicated by the mean DAM. The patch extraction was performed identically to the H-FCN, but the mean DAM was used instead of the discriminative probability map obtained by the statistical test. Moreover, this study indicated that the feature representations extracted from both branches could be complementary, and their fusion could improve classification performance. Although additional information was used to address the shortcomings of H-FCN, the predetermination of patches may hamper the effectiveness of end-to-end learning of local feature extraction and diagnosis. A recent study^30^ introduces a patch-based deep multi-modal learning (PDMML) framework for diagnosing brain diseases. The authors incorporate a discriminative location discovery strategy to remove normal regions without prior knowledge. Moreover, the framework integrates information from different imaging modalities and jointly trains the model to preserve spatial information that would be lost by directly flattening the patches. As a result, the proposed model enhances the accuracy of Alzheimer’s disease diagnosis.

### AD-associated brain-region localization

AD-associated brain-region localization methods have been proposed and developed for various purposes. Specifically, these methods boost the classification performance of diagnostic models, detect potential biomarkers in AD diagnosis, and better explain the behavior of deep learning networks. These brain-region localization methods can be divided into two categories according to the information used in the localization: feature-based and position-based approaches.

First, feature-based approaches produce the brain-region localization result based on individual local features extracted from each brain image. These can be further divided into supervised learning and weakly supervised learning approaches based on the learning strategy. For supervised learning approaches, a relatively large patch or ROI extracted from the image was assigned the same annotation as the image-level annotation^15,48^. A model was trained to represent regional abnormalities by subject. Regional outcomes were used as features to estimate the individual disease states. Although the identified regional abnormalities can provide clinical evidence, the evidence was relatively coarse. In addition, this supervised approach assumes that all regions in patients are affected by AD. This fact has recently led to the application of weakly supervised learning and MIL.

Regarding weakly supervised learning approaches^47^, proposed a representative WSOL method through an FCN with a global average pooling (GAP) layer and linear classifier. Due to the linear property of the GAP and linear classifier, areas that contributed significantly to the predictions can be tracked. This approach has evolved from several perspectives^18,49^, and^50^ proposed bag-of-local-feature models, which provide patch-level class evidence by limiting the receptive field size of the topmost feature maps. In the AD study, WSOL was employed in^26^ to localize AD-related structural abnormalities at a finer scale by training an additional 3D FCN. Moreover, WSOL was applied to represent the regional importance of better feature representation^22,51^. Li et al.^51^ proposed an iterative learning framework leveraged by the localization result generated by WSOL. Further^22^, introduced a subject-specific discriminative brain-region localization called a DAM. An attention mechanism can be attached to a diagnostic model for a similar purpose. Jin et al.^21^ and Zhu et al.^52^ proposed an attention-based diagnosis model for joint learning of discriminative brain-region localization and disease identification.

Unlike feature-based region localization, position-based brain-region localization methods detect regions where significant differences appear between the AD and NC groups. The identified brain regions are consistent across subjects, leading to a method called inter-subject-consistent discriminative region localization^22^. All sMRI scans are aligned to the same template in preprocessing; thus, all samples share the same 3D space. This shared space allows a group comparison of local features, and the statistical test could generate a probability map representing the discriminative capacity. In particular, this position-based localization method has been widely used in patch extraction for patch-level feature representation. Data-driven pathological brain-region localization approaches have continued evolving as described in the previous section such as the statistical approach^25,28^, landmark discovery^25^, pruning strategy^26^, and mean DAM^22^. However, the existing patch extraction methods are performed independently of image-level diagnostic model outcomes.

Inspired by the recent patch-level analysis in AD diagnosis, we propose a framework that jointly learns pathological region localization and disease identification in an end-to-end manner. In addition, final decision-making is conducted through the transparent aggregation of the patch-level responses, providing patch-level class evidence for decision-making. To the best of our knowledge, this framework is the first for joint learning of position-based discriminative brain-region localization and disease identification in an end-to-end manner.

## Experiments and results

In this section, we demonstrate datasets used for performance evaluation, our experimental settings, and comparative methods related patch-level feature extraction. In addition, we report the classification accuracies of our framework and those of comparative methods.

### Dataset and preprocessing

We used two publicly available datasets, namely the ADNI [1] and the AIBL [2]. The ADNI dataset is a renowned and extensively utilized resource in the field of Alzheimer’s disease research. It provides longitudinal data from individuals diagnosed with AD, mild cognitive impairment (MCI), and normal controls (NC) and includes various types of data, such as clinical assessments, neuroimaging scans (i.e., MRI and positron emission tomography (PET)), genetic information, and biomarker measurements. The main objective of the ADNI dataset is to expedite the progress of developing novel diagnostic methods and therapeutic approaches for AD, ultimately contributing to improved patient care. The AIBL database is a comprehensive Australian research initiative focused on investigating the early biomarkers and underlying causes of AD. It consists of a variety of data collected from individuals across the cognitive spectrum, including clinical assessments, neuroimaging scans (MRI and PET), genetics, and lifestyle factors. The dataset provides valuable insight into the development and progression of AD and supports the development of early detection and intervention strategies, and potential treatment approaches. As our main objective is to classify MRI of patients with AD as well as MCI and NC, we consider only baseline subjects. Therefore, we first collected the baseline brain sMRI scans and the diagnostic information from the datasets. Henceforth, we categorized the disease state of scans collected in all datasets (i.e., ADNI and AIBL) into three classes: NC, MCI, and AD. In this process, we further divided each MCI subject into two classes for the MCI conversion prediction task. If the patient corresponding to a baseline image had not been diagnosed with an AD class by 72 months, the image was labeled as the stable MCI (sMCI) class. On the other hand, images converted into the AD class within 36 months were labeled as the progressive MCI (pMCI) class. We noted that MCI samples with reversion from the AD class to other classes were excluded from the dataset. The demographics and clinical information are presented in Table 1.

The collected brain scans were processed using the following pipeline. First, the brain extraction procedure was performed by the HD-BET brain extraction tool^53^ to remove non-brain tissues from the MRI image (e.g., neck, skull, and so on.). Then, skull-stripped images were aligned to the MNI152 template using linear registration tool (FLIRT) from the FMRIB (http://fsl.fmrib.ox.ac.uk/fsl/fslwiki) software library v6.0.1. By doing so, the images removed global linear differences such as global translation, scale, and rotation differences and further allowed them to have an identical spatial resolution (i.e., $1\times 1 \times 1 \,\text {mm}^3$). Consequently, we acquired the preprocessed 3D brain scans with $193 \times 229 \times 193$. Noted that each image was normalized through the mean and standard deviation of each image.

**Table 1:** Demographic information of cohorts included in the study.

Dataset	Category	Gender (male/female)	Age	MMSE	Education
ADNI	NC	214/219	$74.8\pm 5.8$	$29.1\pm 1.1$	$16.2\pm 2.7$
	sMCI	299/198	$72.9\pm 7.7$	$27.8\pm 1.8$	$16.0\pm 2.9$
	pMCI	148/103	$74.1\pm 7.1$	$26.8\pm 1.7$	$15.8\pm 2.8$
	AD	194/165	$75.3\pm 7.9$	$23.2\pm 2.1$	$15.2\pm 3.1$
AIBL	NC	95/107	$73.4\pm 6.4$	$29.0\pm 1.2$	$16.6\pm 2.5$
	AD	91/68	$74.9\pm 8.1$	$23.1\pm 2.1$	$15.7\pm 2.7$

* MMSE* Mini-Mental State Examination.

### Experimental settings

To validate the proposed models, we conducted five-fold cross-validation on the AD diagnosis (AD vs. NC) and MCI conversion prediction (pMCI vs. sMCI). As aforementioned, in order to compare our proposed model with comparative methods in AD diagnosis and MCI conversion prediction tasks, we first trained the model for AD diagnosis and transferred the trained parameters to initialize the network for the MCI conversion prediction task. We performed the transfer learning process because the two tasks are highly correlated, and the MCI conversion prediction task is more challenging than the other task. The classification performances acquired in five-fold cross-validation were measured regarding the accuracy (ACC) and the area under the receiver operating characteristic (AUROC), accordingly.

The proposed method required a predetermined 3D Cartesian space to represent the brain regions where local features were extracted. Therefore, we defined the 3D Cartesian space $\textbf{I}$ with the size of $193 \times 229\times 193 \times 3$ the same as preprocessed 3D brain scans as illustrated in Fig. 2a. To alleviate the overfitting problem, we adopted a random cropping strategy for data augmentation with the size of $177 \times 213 \times 177$ in training. In the case of evaluation, we employed center-cropped images, not only in our proposed method but also in the comparison methods.

Our encoder network consists of a convolutional block, which is set to 32, and four residual blocks, which are set to 32, 64, 128, and 256 the number of output feature maps. In the position-based gating branch, output feature maps for the positional embedding network were set at 128 and 256 and were reduced to 128 and 16 in the gate network. For model initialization, all weights for the AD diagnosis model were initialized using the He initialization method^54^ and optimized using the Adam optimizer^55^. We adopted cosine annealing with the learning rate warm-up method for scheduling the learning rate, referred to by^56^. Specifically, the learning rate was linearly increased from 0 to $1e^{-4}$ within five epochs and was decreased as a cosine function to a learning rate of zero. We set the total number of epochs as 200 and applied the early stopping with 30 patience with four mini-batch sizes. By using a grid hyperparameter search, we set $\lambda$ for the weight between classification and entropy losses as 0.01.

### Comparative methods

For all experiments, we adopted four sMRI-based deep learning architectures, which are considered state-of-the-art methods: 3D CNN, Attention-based 3D ResNet (A3D-Net), DA-MIDL, and HybNet.

- 3D CNN^13^: The CNN-based classifier was trained end-to-end to classify disease states without anatomical prior knowledge and a localization method. We adopted a proposed model architecture for a fair comparison without clinical information, such as patients’ age. Given a randomly cropped 3D image as input, sequential convolutional blocks extracted local features, and the output feature maps were flattened. The flattening vector was employed as input to the classifier. We compared our method to the model trained with a widening factor (WF) of 1 and 2.
- Attention-based 3D ResNet (A3D-Net)^21^: This method had a similar goal as jointly learning AD-related brain-region detection and disease identification in an end-to-end manner. However, there are two underlying differences that exist. One is that the AD-related brain regions were detected based on local features. The other difference is the non-linear interactions between weighted local features for image-level decision-making. An attention module generated spatial attention weights based on local features extracted in the middle of the network, and the attention was applied to local features.
- DA-MIDL^52^: They propose a dual attention network, which represents both spatial importance for extracting discriminative features within each sMRI patch and attention for MIL pooling. The predetermination of patches has been performed by group comparison on the training set. Here, we set the number and size of patches as 60 and $25\times 25\times 25$, respectively. For feature representation, we applied two types of attention. One was spatial attention within each patch, and the other is for attention MIL pooling. An attention-aware global classifier continues to process the bag-level representations for final diagnosis.
- Hybrid network (HybNet)^22^: This method consists of two branches constructed to capture 1) global structural information and 2) local structural information. First, the FCN backbone was trained to generate the DAM and mean DAM to train a GB and LB, respectively. The DAM was directly used as the attention in training the GB, whereas the mean DAM was used to determine the brain regions to extract patches. The LB was trained based on the patches extracted from predetermined brain regions. As described in the literature, we performed the pruning and fine-tuning steps. Finally, two discriminative feature vectors obtained using the GB and LB were concatenated and used as input for the training fusion branch. The fusion branch comprised two subsequent fully connected layers followed by ReLU activation.

**Table 2:** Performance comparison on the ADNI and AIBL dataset.

Methods	AD versus NC (ADNI)		pMCI versus sMCI (ADNI)		AD versus NC (AIBL)	
	ACC	AUROC	ACC	AUROC	ACC	AUROC
3D CNN (WF: 1)^13^	$0.799\pm 0.037$	$0.881\pm 0.034$	$0.689\pm 0.028$	$0.702\pm 0.015$	$0.795\pm 0.029$	$0.884\pm 0.017$
3D CNN (WF: 2)	$0.804\pm 0.014$	$0.886\pm 0.006$	$0.675\pm 0.019$	$0.692\pm 0.024$	$0.698\pm 0.131$	$0.890\pm 0.015$
A3D-Net^21^	$0.827\pm 0.026$	$0.905\pm 0.015$	$0.702\pm 0.029$	$0.720\pm 0.036$	$0.814\pm 0.045$	$0.885\pm 0.016$
DA-MIDL^52^	$0.735\pm 0.040$	$0.809\pm 0.037$	$0.657\pm 0.023$	$0.581\pm 0.019$	$0.832 \pm 0.030$	$0.846 \pm 0.025$
HybNet^22^	$0.832\pm 0.046$	$0.908\pm 0.022$	$0.702\pm 0.014$	$0.676\pm 0.028$	$\mathbf {0.866 \pm 0.054}$	$0.892 \pm 0.043$
BrainBagNet-9	$0.737\pm 0.013$	$0.809\pm 0.029$	$0.643\pm 0.031$	$0.716\pm 0.041$	$0.781 \pm 0.018$	$0.846 \pm 0.012$
FG-BrainBagNet-9	$0.747\pm 0.011$	$0.830\pm 0.021$	$0.643\pm 0.033$	$0.716\pm 0.047$	$0.774 \pm 0.027$	$0.843 \pm 0.024$
PG-BrainBagNet-9	$0.869\pm 0.028$	$0.938\pm 0.025$	$\mathbf {0.715\pm 0.023}$	$\mathbf {0.773\pm 0.009}$	$0.859 \pm 0.014$	$\mathbf {0.928 \pm 0.006}$
BrainBagNet-17	$0.753\pm 0.025$	$0.826\pm 0.022$	$0.655\pm 0.026$	$0.716\pm 0.024$	$0.782 \pm 0.038$	$0.858 \pm 0.018$
FG-BrainBagNet-17	$0.828\pm 0.024$	$0.893\pm 0.022$	$0.679\pm 0.013$	$0.757\pm 0.008$	$0.807 \pm 0.029$	$0.876 \pm 0.013$
PG-BrainBagNet-17	$0.878\pm 0.027$	$\mathbf {0.940\pm 0.017}$	$0.697\pm 0.005$	$0.763\pm 0.010$	$0.832 \pm 0.035$	$0.921 \pm 0.009$
BrainBagNet-25	$0.823\pm 0.017$	$0.891\pm 0.020$	$0.675\pm 0.046$	$0.727\pm 0.032$	$0.767 \pm 0.045$	$0.869 \pm 0.029$
FG-BrainBagNet-25	$0.845\pm 0.025$	$0.909\pm 0.019$	$0.689\pm 0.013$	$0.748\pm 0.013$	$0.801 \pm 0.050$	$0.900 \pm 0.016$
PG-BrainBagNet-25	$0.872\pm 0.025$	$0.939\pm 0.011$	$0.691\pm 0.028$	$0.753\pm 0.031$	$0.822 \pm 0.045$	$0.927 \pm 0.011$
BrainBagNet-41	$0.865\pm 0.043$	$0.929\pm 0.034$	$0.691\pm 0.009$	$0.751\pm 0.026$	$0.840 \pm 0.036$	$0.911 \pm 0.013$
FG-BrainBagNet-41	$0.875\pm 0.030$	$0.920\pm 0.029$	$0.687\pm 0.018$	$0.747\pm 0.016$	$0.799 \pm 0.017$	$0.914 \pm 0.010$
PG-BrainBagNet-41	$\mathbf {0.883\pm 0.013}$	$\mathbf {0.940\pm 0.012}$	$0.695\pm 0.044$	$0.755\pm 0.048$	$0.827 \pm 0.026$	$0.922 \pm 0.014$
BrainBagNet-57	$0.860\pm 0.053$	$0.914\pm 0.031$	$0.683\pm 0.029$	$0.749\pm 0.041$	$0.819 \pm 0.013$	$0.906 \pm 0.015$
FG-BrainBagNet-57	$0.850\pm 0.020$	$0.927\pm 0.023$	$0.701\pm 0.019$	$0.764\pm 0.017$	$0.836 \pm 0.020$	$0.920 \pm 0.006$
PG-BrainBagNet-57	$0.862\pm 0.026$	$0.937\pm 0.020$	$0.681\pm 0.021$	$0.755\pm 0.015$	$0.841 \pm 0.012$	$0.923 \pm 0.009$

**Figure 3:**
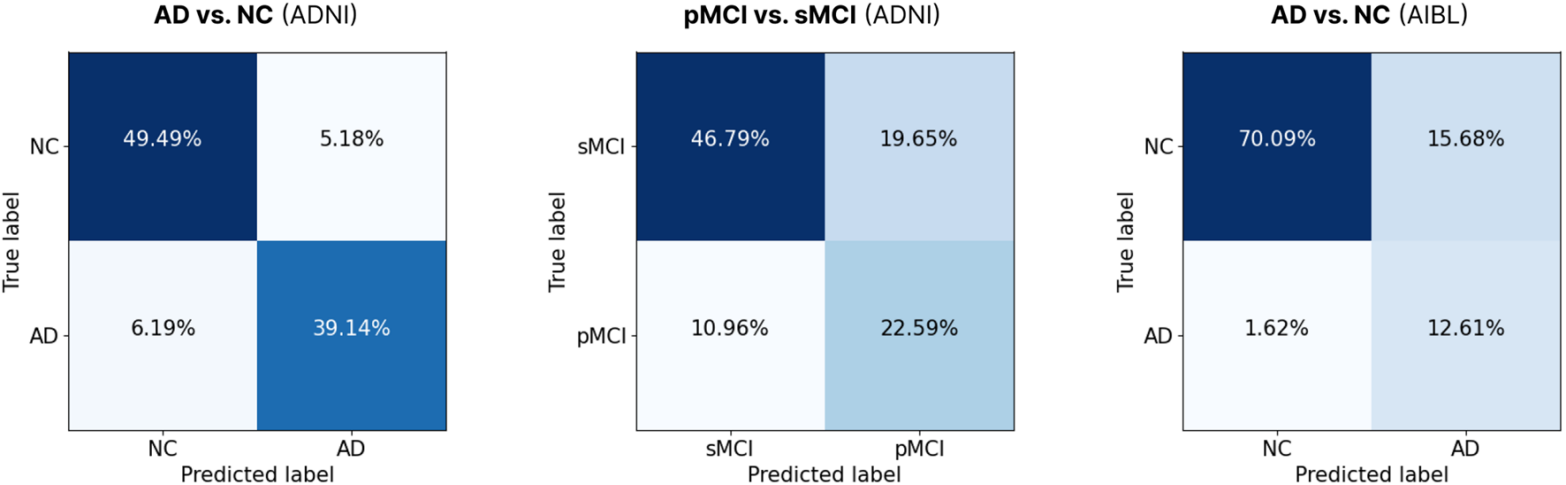
Confusion matrices derived from the PG-BrainBagNet-41 and PG-BrainBagNet-17 models, which demonstrated the best performance based on the validation loss criterion, for the tasks of AD diagnosis and MCI conversion prediction, respectively. Each confusion matrix depicts the performance of the respective model on its corresponding task and dataset.

### Performance comparison

Table 2 represent the comparison of classification performance for the AD diagnosis and MCI conversion prediction task, respectively. In the table, BrainBagNet-*s* processes a single patch size *s*, and patch-level responses are aggregated using the GAP operation. The FG-BrainBagNet indicates a BrainBagNet with a feature-based gate inspired by an attention-based MIL framework^38^. Instead of using position information, local features generated by the encoder network were used as input to the gate network. Lastly, the PG-BrainBagNet is the proposed position-based gate method.

In the AD diagnostic task, we first selected the patch size as a tunable hyperparameter using the mean balanced cross-entropy loss criterion of the validation set for five-fold cross-validation. The best classification model in AD diagnostic task was selected as PG-BrainBagNet-41 based on the minimum validation loss. Compared to state-of-the-art methods, we observed that our proposed method outperformed in terms of accuracy (ACC) and AUROC. The highest and lowest margins for the mean accuracy were $14.77\%$ (vs. DA-MIDL) and $3.65\%$ (vs. HybNet (GB)), respectively. The proposed position-based gate method (i.e., PG-BrainBagNet) yields an increase in classification performance compared to BrainBagNets regardless of patch size. Moreover, PG-BrainBagNet significantly improved the classification performance of BrainBagNet (i.e., 13.13%) with small patch size (i.e., s=9), whereas feature-based gates (i.e., FG-BrainBagNet) even had low performance when $s=57$. The proposed position-based gate method (i.e., PG-BrainBagNet) yields an increase in classification performance compared to BrainBagNets regardless of patch size. Moreover, PG-BrainBagNet significantly improved the classification performance of BrainBagNet (i.e., 13.13%) with small patch size (i.e., s=9), whereas feature-based gates (i.e., FG-BrainBagNet) even had low performance when $s=57$. Although the classification performance of BrainBagNets increased as the patch size increased, PG-BrainBagNets did not exhibit significant differences according to patch size. Therefore, the reason that BrainBagNets performed classification poorly when using a small patch size might be that the whole-brain image contains many patches unrelated to the brain disease. In addition, we observed that the improvement in classification performance cause our proposed method (w/ gating mechanism) to capture the AD-related regions effectively. For validating the generalization of the models trained on the ADNI dataset, we additionally tested on the AIBL cohort. We could observe that the position-based gating branch (i.e., PG-BrainBagNet) improved the accuracy and AUROC score. In addition, our proposed method showed a balanced prediction result even in the case of class imbalance. Although HybNet achieved the highest accuracy, our proposed model obtained more balanced performance, outperforming the state-of-the-art models in terms of AUROC.

In the MCI conversion prediction task, the best model that appeared in our proposed method was PG-BrainBagNet-17 based on the minimum validation loss as well. In the comparison of the results using the state-of-the-art methods, the classification accuracy did not show a significant difference due to the class-imbalanced dataset which consists of the number of samples 251 (pMCI) and 497 (sMCI). On the other hand, we could observe that our proposed method significantly increased the AUROC score against the comparison method. Similar to the performance obtained in the aforementioned task, in terms of patch sizes, the classification performance of BrainBagNets increased as the patch size increased excluding $s=57$. Furthermore, the feature-based gating method (i.e., FG-BrainBagNet) did not play a role in improving the classification results. However, the position-based gate method (i.e., PG-BrainBagNet) yielded improvements when a small patch size was used, especially when the patch size was 9, 17, 25 or 41. Except for $s=57$ case, the classification results of PG-BrainBagNet were consistently increased as the patch size was reduced. As the receptive field size was limited, local feature representations were forced to extract the local brain changes rather than global structural changes. The results implied the brain-region localization method based on the position provided highly informative results. In addition, we observed the importance of capturing subtle changes for the early detection of AD.

The MCI conversion prediction performance obtained from the proposed models trained from scratch and those trained through transfer learning is compared in supplementary A. Furthermore, Fig. 3 illustrates the confusion matrices of the PG-BrainBagNet-41 and PG-BrainBagNet-17 models, which exhibited the best performance according to the validation loss criterion, for the tasks of AD diagnosis and MCI conversion prediction, respectively. Each confusion matrix showcases the performance of the corresponding model on its respective task and dataset.

**Figure 4:**
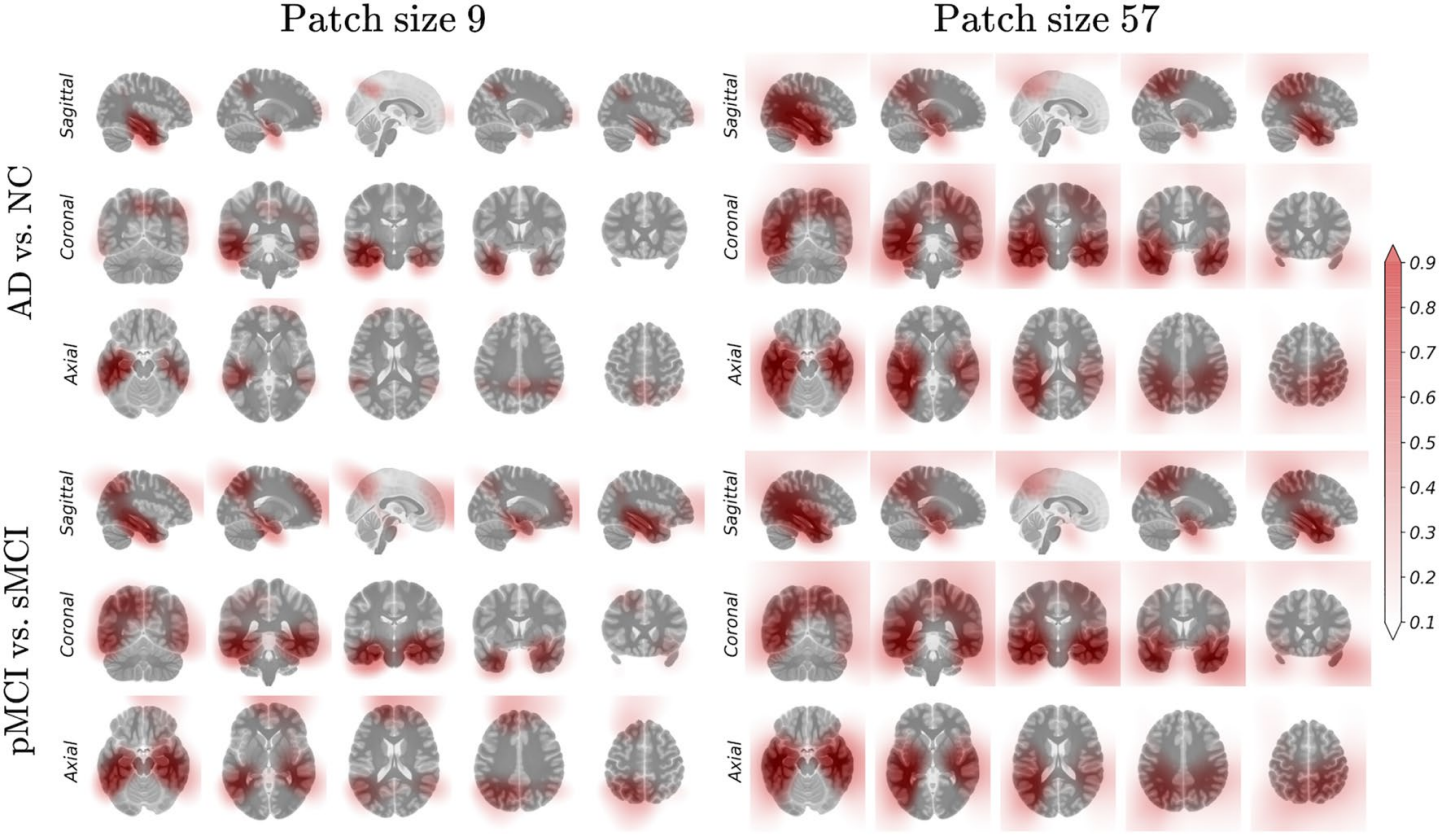
Difference in the output of the trained position-based gating branch depending on the downstream task and patch size used in model training.

### Visualization of discriminative brain regions

We analyzed discriminative probability maps $\textbf{G}$ generated by the proposed position-based gating branch. For better visualization, linear interpolation was performed and overlaid with the MNI template. The changes in the discriminative probability map by the learning epoch are described in supplementary B. In the learning process with a small patch size, localization helped the diagnostic model extract finer features; thus, the gating branch could provide better localization results based on the features extracted by the diagnostic model. The differences in the output of the gating branch from the trained model according to the patch sizes and downstream tasks are compared in Fig. 4. First, high responses were distributed in anatomically meaningful areas such as the hippocampal, temporal, and parietal lobe areas. When the model was limited to increasing the receptive field size, the gate network represented the weight in the sparse regions only. As the patch size increased, regions with high responses were captured in the overall images. In this context, the proposed method employing small patches is sensitive to localization results and requires proper localization. While there were no significant changes by downstream tasks, we observed that the highlighted regions were dispersed especially in the model trained using small patches for MCI conversion prediction.

**Figure 5:**
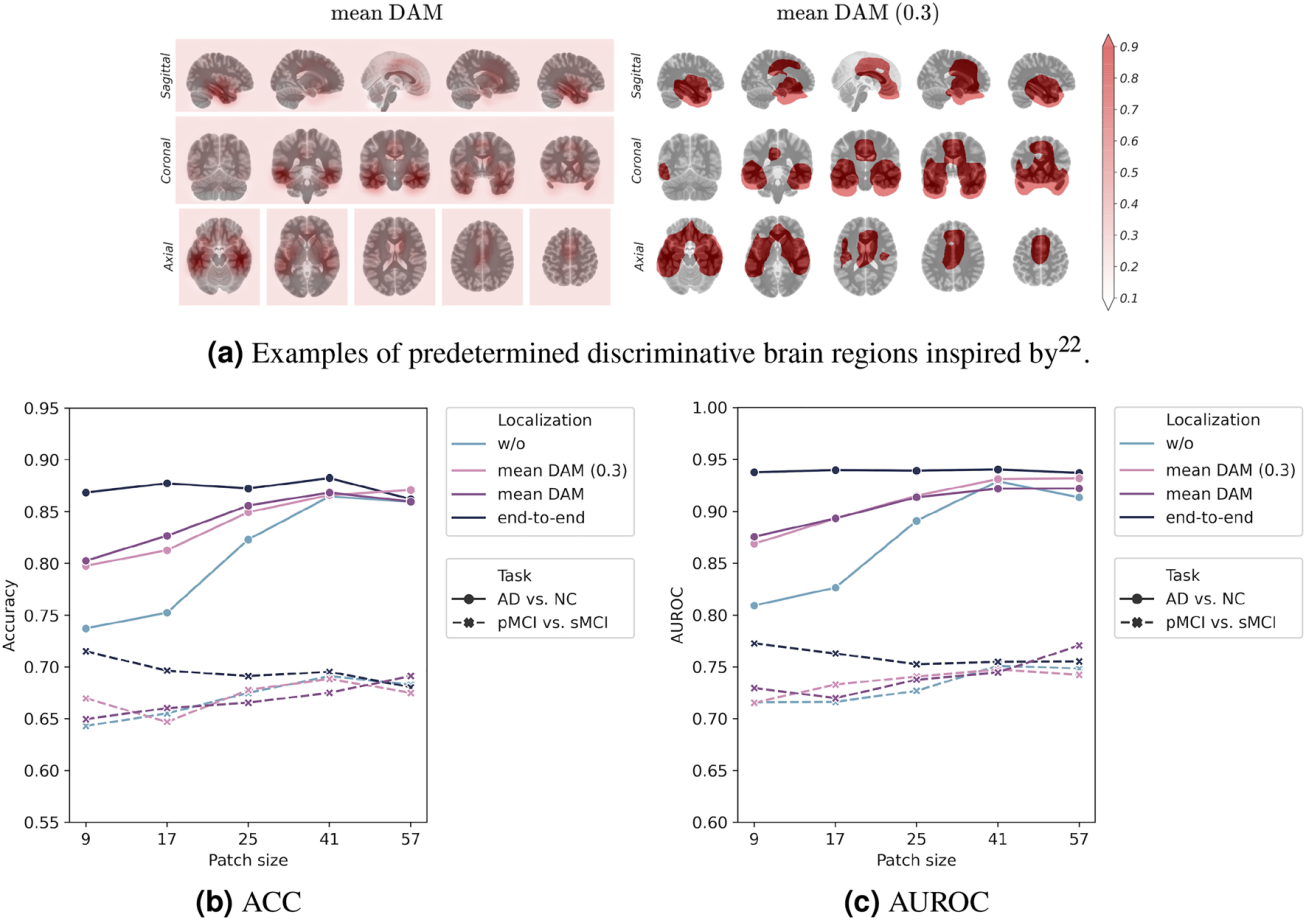
(**a**) Examples of predetermined discriminative brain regions inspired by^22^. (**b**) ACC. (**c**) AUROC. Illustration of classification performance in terms of accuracy (ACC) and area under the receiver operating characteristic (AUROC) by the patch size used in model training, localization method, and downstream task.

### Effect of brain region localization on diagnostic performance

We performed an ablation study of localization methods using our proposed framework to evaluate the effectiveness of joint learning of discriminative brain-region localization and disease identification. We compared four localization methods: “w/o”, “mean DAM”, “mean DAM (0.3)”, and “end-to-end”. The “w/o” denotes BrainBagNets, and the “end-to-end” denotes the proposed models, which are PG-BrainBagNets. Both “mean DAM” and “mean DAM (0.3)” were models trained with the predetermined discriminative brain region inspired by the mean DAM introduced in^22^. For “mean DAM”, the proposed framework has been trained using predetermined $\textbf{G}$ by considering the mean DAM to be $\textbf{G}$ instead of training position embedding and gate network. The resulting model was denoted as “mean DAM”. In addition, we generated a binary mask because “mean DAM” was not used as a probabilistic value but instead was used for extracting patches in^22^. In patch extraction, the threshold of 0.3 was used in the literature to represent potential patch locations. We obtained a binary mask based on this threshold, and model training was performed in the same way as for “mean DAM”. The predetermined $\textbf{G}$ for the “mean DAM” and “mean DAM (0.3)” are illustrated in Fig. 5a.

The average accuracy (ACC) and AUROC in five-fold cross-validation are described in Fig. 5b and c. First, when the model was trained without brain-region localization, classification performance decreased as the patch size reduced. The model trained using the smallest patches exhibited the lowest classification performance for both tasks in accuracy and AUROC. By adding the predetermined localization method, the classification performance improved compared with that without the localization method. However, localization was performed regardless of the diagnosis model, resulting in a worse classification than the proposed method (i.e., “end-to-end”). In the MCI conversion prediction task, only the proposed method demonstrated increased classification performance by limiting the increase in patch size. This result implies that the regularization of the patch size allows extracting AD-related local and subtle changes but requires suitable brain-region localization dependent on the diagnosis model.

**Figure 6:**
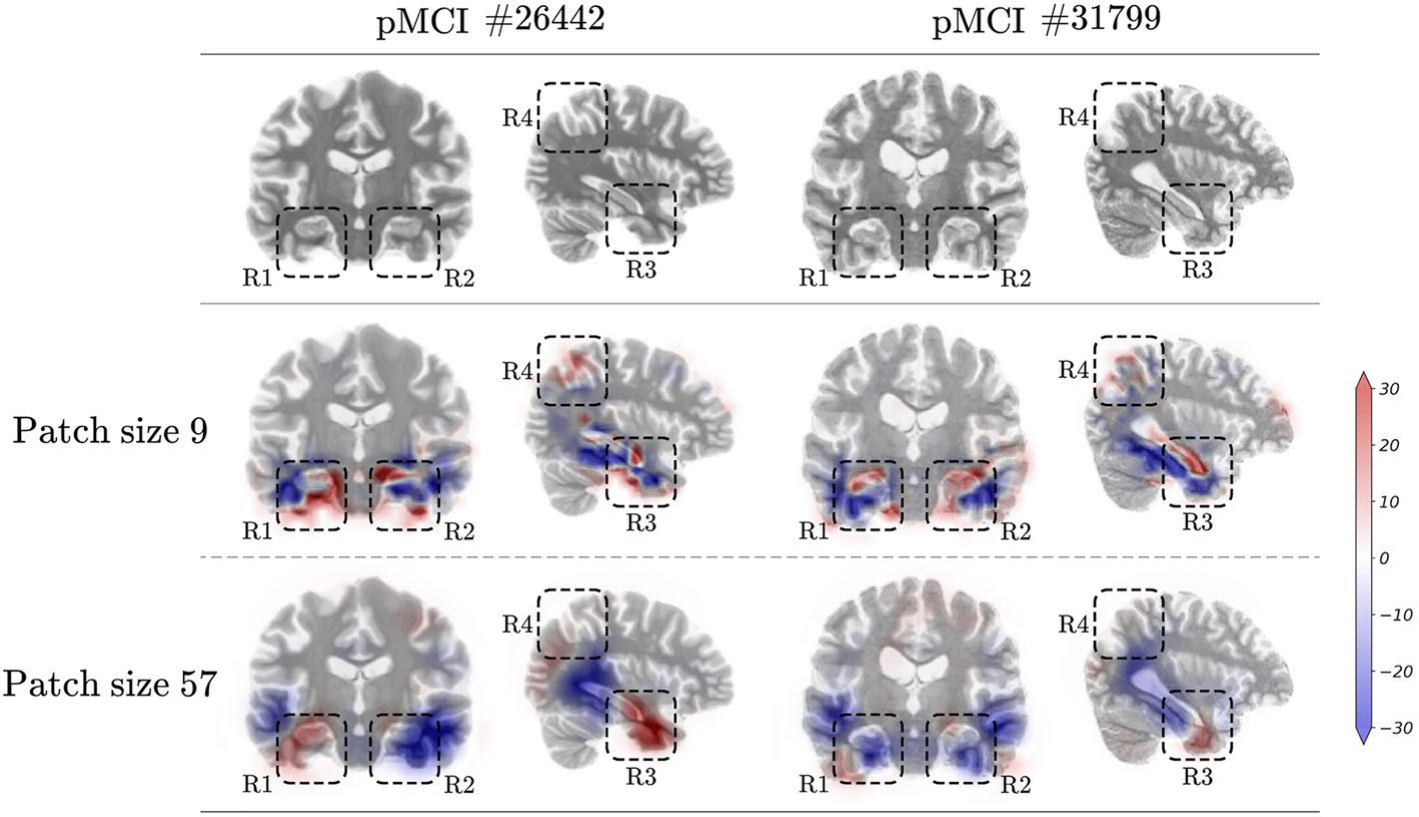
Examples of false negatives from the model trained with large patches but correctly predicted by the model trained using small patches. Each column indicates one sample labeled as progressive mild cognitive impairment (pMCI), where the number next to the # denotes the corresponding image ID of an input MRI scan. In addition, the blue and red colors indicate high class evidence for the stable mild cognitive impairment and pMCI class in that region, respectively.

### Effectiveness of subtle changes captured using small patches

The aforementioned, we demonstrated that the model extracting the local class evidence captured using a small receptive field size better predicts MCI conversion. We analyzed the two pMCI samples that yielded false negatives from the model trained using a large patch and made predictions correctly by limiting the patch size. The local class evidence according to patch size is described in Fig. 6. The first row depicted the original sMRI scans with image ID 26442 and 31799. The following rows demonstrated the patch-level class evidence according to the patch size. For a better comparison, the areas where a high amount of class evidence was contained were marked with dashed rectangles and denoted as R1 to R4. The blue and red colors indicate high-class evidence for sMCI and pMCI classes in that region, respectively.

First, in the bottom of the R1 region, we observed that Sample #26442 contains brain atrophy in the temporal lobe rather than the hippocampus, compared to Sample #31799. In contrast, Sample #31799 depicts brain atrophies located in the hippocampal area. These brain atrophies were correctly captured by the model trained using small patches. However, the estimation produced by the model trained using larger patches demonstrated the difficulties of capturing these subtle changes. These patterns can be observed in the R1, R2, and R3 regions. The positive class evidence for the pMCI class located in the parietal lobe area was only captured by the model trained using small patches, which can be observed in comparing the R4 region. Finally, the model trained using small patches could determine sufficient local evidence to correctly predict the MCI conversion, whereas models trained using large patches could not capture sufficient cues for a correct decision. In this analysis, we observed that regularizing the increasing patch size increased the prediction performance for MCI conversion by extracting subtle and local structural feature representation. To better understand these results, we demonstrated the additional visualization of patch-level class evidence for the various patch sizes and samples in supplementary C.

## Discussion

From a practical perspective, there are several pros and cons to consider in our study. On the positive side, our proposed framework deviates from previous patch-level approaches by leveraging a CNN-based encoder to extract patch-level features directly from whole-brain images, leading to improved computational efficiency. This efficiency is beneficial for practical implementation and real-time applications. However, there are some limitations to acknowledge. First, our study primarily focused on the early-stage diagnosis of MCI and does not address the forecasting of disease progression. Future research is needed to extend the framework capabilities to predict the progression of brain diseases. Moreover, while our proposed framework provides explainability by visualizing a probability map to aid clinicians and patients in understanding the models’ decisions, estimating decisions uncertainty remains an important consideration that requires further exploration. Incorporating uncertainty estimation into the framework would enhance its reliability and trustworthiness. Lastly, given the recent advancements in multi-modal learning, it would be worthwhile to explore the integration of other neuroimaging modalities, such as PET and diffusion-weighted imaging, to improve the overall performance and diagnostic accuracy of our current framework.

## Conclusion

In this work, we proposed a deep learning train pipeline for patch-level feature representation learning on MRI scans. To alleviate the problem caused by predetermined brain regions for patch-level feature representation learning, we proposed a PG-BrainBagNet framework for jointly learning discriminative brain-region localization and disease identification in an end-to-end manner. We conducted both the AD diagnosis and MCI conversion prediction tasks on two publicly available datasets, thereby demonstrating the validity of our proposed method. Specifically, our PG-BrainBagNet obtained the best classification performance over competing methods. Furthermore, our PG-BrainBagNet effectively increased the classification performance, when localization of the subtle changes was required. We also demonstrated the interpretability of the proposed method by tracing the rationale for the model predictions down to the small patch level.

## Methods

We propose PG-BrainBagNet as depicted in Fig. 1, wherein our proposed framework consists of four kinds of networks: the encoder, classifier, position embedding, and gate network. Specifically, these networks are organized into two branches such as patch-level prediction and position-based gating and each branch takes different inputs: the input MRI scan $\textbf{X}$ and the position indicator $\textbf{I}'$. The input MRI scan $\textbf{X}$ can be considered a set containing *M* patches $\textbf{X} = \{\textbf{x}_1,\cdots ,\textbf{x}_M\}$. $\textbf{I}'$ represents the patch position information: the position indicator. In the following section, we present the details for jointly learning AD-related local morphological changes and the regions where the discriminative changes sustainably appear based on the branches.

### Patch-level features extraction and classification

The patch-level prediction branch comprises an encoder $\mathscr {E}^s_{\phi }$ and classifier network $\mathscr {C}_{\psi }$ parameterized with $\phi$ and $\psi$, respectively. First, according to the receptive field size of the top-level feature maps, we construct an encoder network that can adjust the patch size for feature extraction to handle local and morphological changes distributed in the whole brain. The configured encoder takes the whole brain image $\textbf{X} \in \mathbb {R}^{W\times H\times D \times 1}$ as input and extracts the local features from 3D patches of size $s\times s\times s$, where *W*, *H*, and *D* denote the size of images’ width, height, and depth, respectively. The patch-level features, extracted from the whole brain, are represented as feature maps $\hat{\textbf{X}} \in \mathbb {R}^{w\times h\times d\times f}$, where *w*, *h*, and *d* denote the size of 3D spatial dimension, and *f* is the size of the feature space. In particular, the kernel and stride sizes in the convolution operator allow adjusting the patch sizes and the distance between them. Furthermore, we employed this approach introduced in BagNets^18^ to construct our proposed network architectures. Specifically, based on BagNets, we rebuilt shallower encoders utilizing 3D convolutional layers. The goal of configuring an encoder network $\mathscr {E}^s_{\phi }$ is to represent local features extracted from patches of size $s \times s\times s$.

Specifically, encoder network $\mathscr {E}^s_{\phi }$ consists of a convolutional block, max-pooling, and four residual blocks, as illustrated in Fig. 1. All convolutional blocks in this study include sequential operators of the convolutional layer, instance normalization layer, and rectified linear unit (ReLU) activation function. The kernel and stride size for the first convolutional block is set to $5\times 5\times 5$ and $2\times 2\times 2$, respectively. For the following max-pooling layer, the kernel size is $3\times 3\times 3$, and the stride size is $2\times 2\times 2$. The feature maps yielded by max pooling have a receptive field size of $9\times 9\times 9$. Thus, if the receptive field size does not increase further, the encoder can extract features with a specific receptive field size of $9\times 9\times 9$ from the whole brain. With nine as the minimum size, we constructed encoders based on five receptive field sizes according to the following residual blocks.

Overall, we can achieve patch-level feature representation $\hat{\textbf{X}}\in \mathbb {R}^{w \times h \times d \times f}$ for an individual MRI scan $\textbf{X}$. Then, the classifier network $\mathscr {C}_{\psi }$ converts the *f*-dimensional vector into a scalar for patch-level responses and produces $\tilde{\textbf{X}} \in \mathbb {R}^{w \times h \times d \times 1}$. The patch-level responses $\tilde{\textbf{X}} = (\tilde{x}_{1,1,1},\cdots ,\tilde{x}_{i,j,k},\cdots ,\tilde{x}_{w,h,d})$ comprises local responses $\tilde{x}_{i,j,k} \in \mathbb {R}$. As both the encoder and classifier networks extract local responses in the specific receptive field size and share the extracting function overall spatial dimensions, each local response was extracted by a 3D patch in the brain without considering its position in the brain.

### Position-based gate for AD-related brain-region localization

This branch is to represent the probability of detecting AD-related morphological changes in patches centered on specific coordinates in MR scans. As all MRI scans were aligned in a 3D template in the image processing step, a 3D space can be shared and is applicable over the samples. In addition, all 3D patches are single-scale patches with 3D cubic shapes. Thus, the patches distributed in the entire brain can be differentiated only by the patch position information, and the center patch position is the representative position information. The simplest method to indicate positions is to use a one-hot representation. However, this approach can be inefficient due to the numerous patches and ignores the volumetric position in 3D space. This problem can be efficiently addressed using the Cartesian coordinate system.

Inspired by a representation proposed in^57^, we constructed a 3D complete translation invariance to specify a 3D Cartesian space. The 3D Cartesian space coordinates could be represented in three channels, such as $\textbf{I} \in \mathbb {R}^{W'\times H'\times D' \times 3}$.

Based on the position information, we obtain the center position information for the patches used in the patch-level prediction branch. The center position information is represented as a position indicator $\textbf{I}'$. The extraction of the position indicator is described in Fig. 2b. First, when using data augmentation such as image translation and cropping, the representation of 3D Cartesian space $\textbf{I}$ should be transformed in the same way as the input transformation, which results in the same spatial dimension as the input MRI scan. Then, based on the encoder network, the center positions of the receptive field are hierarchically extracted. The extracted position indicator $\textbf{I}'\in \mathbb {R}^{w\times h\times d\times 3}$ is taken as input for the position-based gating branch.

The position-based gating branch generates translation-dependent outcomes, which is not possible in the patch-level prediction branch. As described in Fig. 1, parameterized functions in position embedding $\mathscr {P}_{\pi }$ and gate network $\mathscr {G}_{\rho }$ consist of convolutional layers, and all convolutional layers in both networks are point-wise convolutions parameterized by $\pi$ and $\rho$. In the position embedding network, the semantic feature representation $\hat{\textbf{I}}$ were extracted to detect the task-oriented discriminative region by increasing the number of feature maps. Furthermore, the number of output feature maps was decreased in the gate network to encode the semantic feature representation. Finally, the remaining feature maps were averaged and activated by the sigmoid activation function to generate discriminative probability map $\textbf{G} = (g_{1,1,1},\cdots ,g_{i,j,k},\cdots ,g_{w,h,d})$. The discriminative probability map consists of $g_{i,j,k}\in [0, 1]$, representing the position-based response located in (*i*, *j*, *k*). By constructing position indicator $\textbf{I}'$ based on the representation of coordinates in the 3D Cartesian space, absolute positioning can be performed and shared over the MRI scans for each patch. Therefore, the trained position embedding and gate network represent the high response in the region where the AD-related morphological changes are consistently captured.

### Gate-based pooling for image-level prediction

By considering a 3D whole brain to be a bag and considering the local features extracted from 3D patches distributed in the whole brain to be instances, the proposed framework can be considered a MIL framework. In conventional MIL-based classification problems, permutation-invariant pooling operators (e.g., max and mean) have been widely used to aggregate instance-level representation into bag-level representation. Just as^18^ introduced GAP for patch-level responses in aggregation, the mean operator has also been used as a representative aggregation function, especially when more than one instance is needed to identify a bag. The mean operation can directly calculate the image-level response, *z*, as follows:

1 $$\begin{aligned} z = \frac{1}{whd} \sum ^{w,h,d}_{i,j,k=1} \tilde{x}_{i,j,k}. \end{aligned}$$

A function parameterized by neural networks was proposed in^38^ to detect key instances and aggregate the responses based on them. Inspired by the aggregation method, we defined the image-level response by aggregating patch-level responses through position-based outcomes of the gate network. The element-wise multiplication between patch-level responses $\tilde{\textbf{X}}$ and discriminative probability map $\textbf{G}$ results in patch-level class evidence $\textbf{E}\in \mathbb {R}^{w\times h\times d\times 1}$. The total amount of the discriminative brain region is unknown; thus, the normalization is performed based on the sum of the discriminative probability map so that the amount of the gated regions is independent of the diagnostic results. The aggregation of patch-level class evidence infers image-level abnormality and is defined as follows:

2 $$\begin{aligned} z = \frac{1}{\sum ^{w,h,d}_{i,j,k=1} g_{i,j,k}} \sum ^{w,h,d}_{i,j,k=1} e_{i,j,k}, \end{aligned}$$

where $e_{i,j,k} = g_{i,j,k}\tilde{x}_{i,j,k}$. The image-level response *z* is directly activated by the posterior probability $\hat{y} = p(y|\textbf{X})$ using the sigmoid activation function. The patch-level class evidence $\textbf{E}$ directly reveals which patches made a significant contribution in the final decision, making the model transparent and interpretable.

### Joint learning of pathological brain-region localization and disease identification

The overall parameters (i.e., $\phi$, $\psi$, $\pi$, and $\rho$) are trained based on the image-level classification objective. To better train from the generalization perspective, the proposed models were trained using two additional techniques: label smoothing and balanced cross-entropy, referring to prior studies^56,58,59^. The classification loss function is described as follows:

3 $$\begin{aligned} \mathscr {L}_{\text {cls}} = -\beta y^{LS}\text {log}\hat{y}-(1-\beta )(1-y^{LS})\text {log}(1-\hat{y}), \end{aligned}$$

where $y^{LS} \in \{0.1, 0.9\}$ is the modified target and $\beta$ is a hyperparameter addressing the imbalanced classification problem. In addition, $\beta$ was set to the inverse class frequency. Precisely, the function was calculated using the number of samples with negative annotation ($y=0$) divided by the total number of samples. The gradient generated by classification loss updates the parameters, $\phi$, $\psi$, $\pi$, and $\rho$.

Moreover, the element-wise multiplication operation between $\textbf{G}$ and $\tilde{\textbf{X}}$ allows both forward and backward propagation to be highly dependent on each other. However, in the early stages of training, randomly initialized parameters yielded both $\tilde{\textbf{X}}$ and $\textbf{G}$. To impose the framework to explore more discriminative brain regions localization, we employ an entropy loss for maximization of entropy $\textbf{G}$, as follows:

4 $$\begin{aligned} H(\textbf{G})= & {} - \frac{1}{whd}\sum ^{w,h,d}_{i,j,k=1}( {g_{i,j,k}}\text {log}{g_{i,j,k}} +(1-g_{i,j,k})\text {log}(1-g_{i,j,k}) ), \end{aligned}$$

5 $$\begin{aligned} \mathscr {L}_{\text {ent}}= & {} - H(\textbf{G}). \end{aligned}$$

The gradient generated by the entropy loss is affected on parameters $\pi$ and $\rho$. The final total loss function is defined using hyperparameter $\lambda$ to weigh the classification loss and entropy loss. Our proposed network is trained in an end-to-end manner with the following loss function:

6 $$\begin{aligned} \mathscr {L}_{\text {total}} = \mathscr {L}_{\text {cls}} + \lambda \mathscr {L}_{\text {ent}}. \end{aligned}$$

## Supplementary Information

Supplementary Information.

## Data Availability

We have evaluated our proposed method on the Alzheimer’s Disease Neuroimaging Initiative (ADNI) and the Australian Imaging, Biomarker & Lifestyle Flagship Study of Ageing (AIBL) dataset. Both datasets are publicly available, and more information can be found at the following link: (ADNI) https://adni.loni.usc.edu/data-samples/access-data/, (AIBL) https://aibl.csiro.au/.

## Code availability

All codes used in our experiments are available at https://github.com/ku-milab/PG-BrainBagNet.
