# Supplementary material for: Deep joint learning of pathological region localization and Alzheimer’s disease diagnosis
Source: Sci Rep. 2023 Jul 19;13:11664. doi: 10.1038/s41598-023-38240-4 (PMC10356790; doi:10.1038/s41598-023-38240-4)
Supplement: Supplementary file 1 — Supplementary Information. [file 41598_2023_38240_MOESM1_ESM.pdf]

Deep Joint Learning of Pathological Region Localization and Alzheimer's Disease Diagnosis

Changhyun Park[1,+], Wonsik Jung[1,+], and Heung-Il Suk[1,2,*]

[1]Department of Brain and Cognitive Engineering, Korea University, Seoul 02841, Republic of Korea

[2]Department of Artificial Intelligence, Korea University, Seoul 02841, Republic of Korea

[+]These authors contributed equally to this work.

[*]Corresponding author: hisuk@korea.ac.kr

Supplementary A. Transfer Learning for Mild Cognitive Impairment (MCI) Conversion Prediction

Due to the high correlation between Alzheimer's disease (AD) diagnosis and MCI conversion prediction, the more challenging MCI conversion prediction task was aided by knowledge learned from subjects with AD and normal control (NC). To confirm the effect of transfer learning, we compared two types of models for the MCI conversion prediction task: those trained from scratch and those trained through a transfer learning strategy using parameter initialization with the AD diagnostic model. For each experimental setting (from scratch vs. transfer learning), we trained models based on the BarinBagNet (w/o position-based gating branch) and PG-BrainBagNet (w/ position-based gating branch). The result is described in Table S.1. We observed that all models obtained through transfer learning had better classification performance in terms of area under the receiver operating characteristic (AUROC) than models trained from scratch.

Table S.1. Effectiveness of transfer learning in MCI conversion prediction tasks (progressive MCI vs. stable MCI) by comparing the five-fold classification accuracy (AUROC).

Model		Learning from scratch	Transfer learning
		ACC (AUROC)	ACC (AUROC)
BrainBagNet (w/o position-based gating branch)	Patch 9	0.635 (0.708)	0.643 (0.716)
	Patch 17	0.628 (0.702)	0.655 (0.716)
	Patch 25	0.635 (0.691)	0.675 (0.727)
	Patch 41	0.644 (0.704)	0.691 (0.751)
	Patch 57	0.643 (0.731)	0.683 (0.749)
PG-BrainBagNet (w/ position-based gating branch)	Patch 9	0.690 (0.761)	0.715 (0.773)
	Patch 17	0.682 (0.748)	0.697 (0.763)
	Patch 25	0.671 (0.739)	0.691 (0.753)
	Patch 41	0.682 (0.737)	0.695 (0.755)
	Patch 57	0.690 (0.735)	0.681 (0.755)

Supplementary B. Jointly Learning Discriminative Brain Regions

Previous studies used predetermine brain regions to extract patches before learning patch-level feature representation. This problem could have been effectively mitigated by our proposed framework, jointly learning for discriminative brain-region localization and brain disease prediction in an end-to-end manner. In Fig. S.1, the changes of the localization results by learning epoch have been shown. The red color indicates a high response from the gate network. We could observe the different visualization results by patch size used in the diagnostic model. It implicates the optimal localization result is dependent on the diagnostic model.

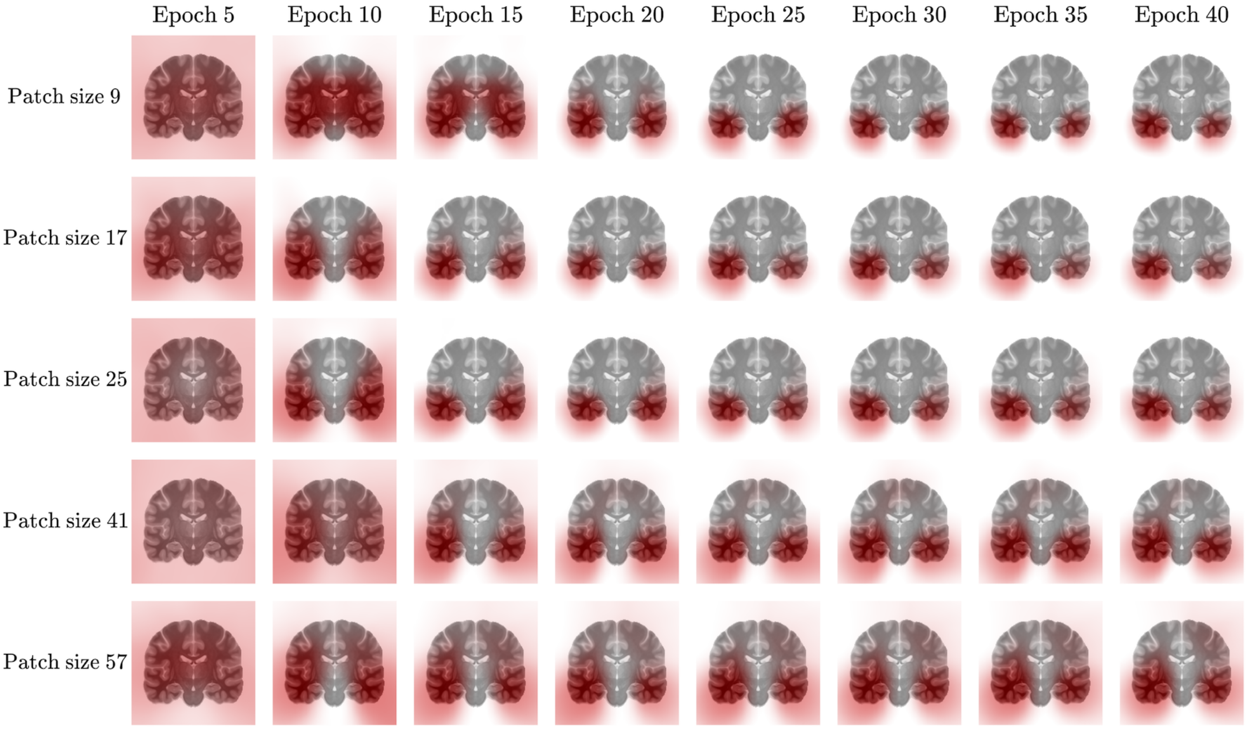

Figure S.1. Changes in the output of the proposed position-based gating branch according to the patch size used in model training and the number of training epochs. The red color indicates a high response from the gate network.

Supplementary C. Visualization of Patch-level Class Evidence

Our proposed method performed image-level decision-making by aggregating patch-level class evidence. The patch-level class evidence produced by samples that yielded true positives and false positives predicted with high confidence is demonstrated in Fig. S.2.

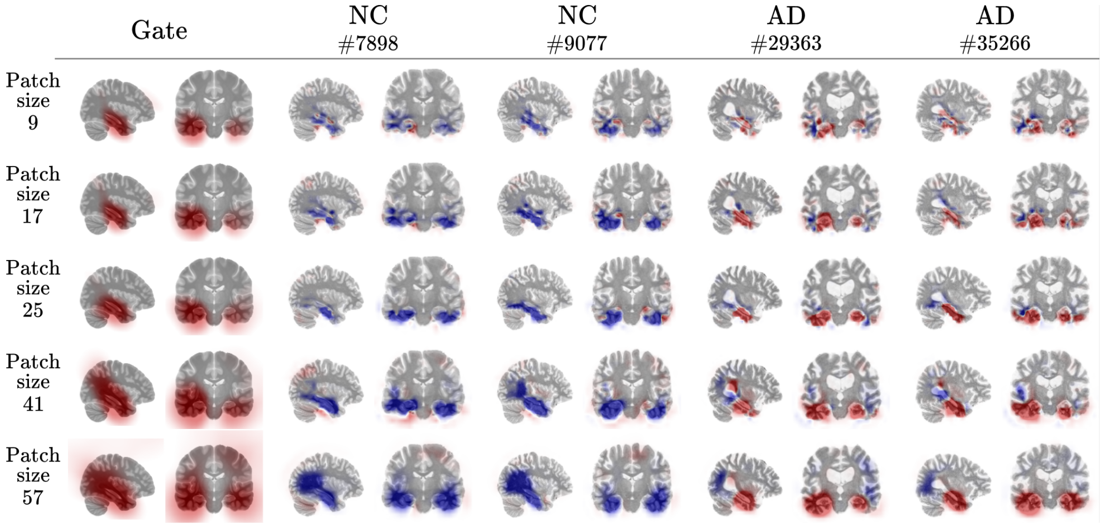

(a) AD vs. NC.

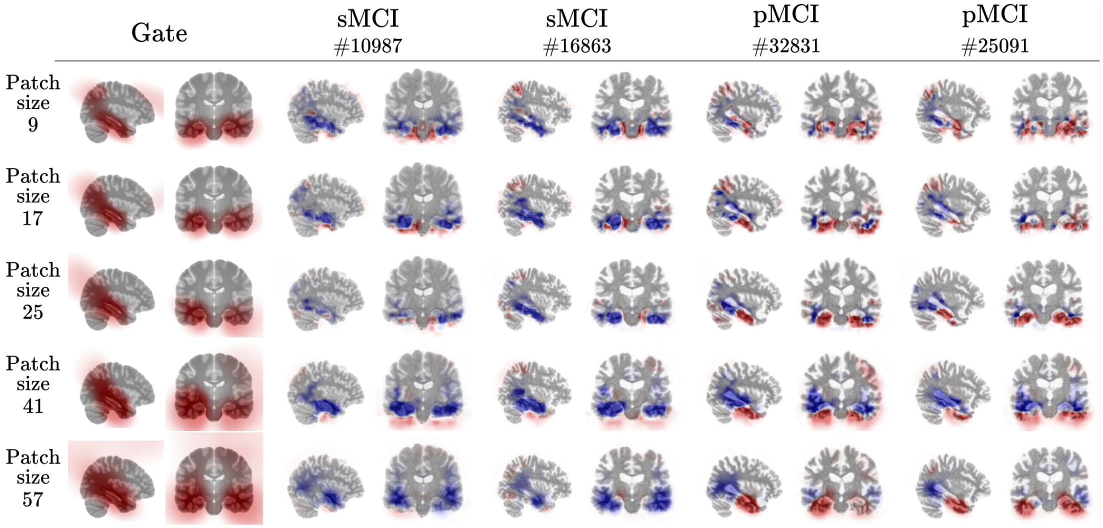

(b) pMCI vs. sMCI.

Figure S.2. Illustration of gating result and patch-level class evidence for samples correctly predicted with high confidence. Each column indicates one sample labeled as NC, sMCI, pMCI, and AD, where the number next to the # denotes the corresponding image ID of an input MRI scan. In addition, the blue and red color indicate high class evidence for the negative (i.e., NC and sMCI) and positive (i.e., AD and pMCI) class in the patches centered on that region, respectively.
